# Effective adsorption of fluorescent congo red azo dye from aqueous solution by green synthesized nanosphere ZnO/CuO composite using propolis as bee byproduct extract

**DOI:** 10.1038/s41598-024-58306-1

**Published:** 2024-04-20

**Authors:** Ahmed Radwan, Samir O. Mohamed, Mostafa M. H. Khalil, Islam M. El-Sewify

**Affiliations:** 1 Department of Chemistry, Faculty of Science, Ain Shams University, Abbassia, Cairo 11566 Egypt; 2 Physics Department, Faculty of Science, Ibb University, Ibb, Yemen; 3 Engineering College, Al Janad University for Science and Technology, Taiz, Yemen

**Keywords:** Nanocomposite, Green, Removal, Adsorption capacity, Wastewater, Nanoscale devices, Environmental sciences, Chemistry, Nanoscience and technology

## Abstract

The indirect dumping of massive volumes of toxic dyes into water has seriously affected the ecosystem. Owing to the many applications of the designed nanomaterials in the manufacturing process, there is a lot of research interest in synthesizing nanomaterials using green processes. In this research, the byproduct of bee was employed to synthesize nanoparticles (NPs) of ZnO, CuO, and biosynthesized ZnO/CuO (BZC) nanocomposite via utilizing a green and simple approach. To validate the effective fabrication of BZC nanocomposite, various characterization measurements were applied. FTIR analysis identified the functional groups in charge of producing nanoparticles and nanocomposites. Moreover, the existence of ZnO and CuO XRD peaks suggests that the nanocomposites were successfully biosynthesized. The high-resolution XPS spectrum of the BZC nanocomposite’s Zn2p3, Cu2p3, and O1s were observed. Our findings indicate the successful engineering of the prepared nanomaterials and BZC nanocomposite. Our findings indicate the successful engineering of the prepared nanomaterials and BZC nanocomposite. For Congo red (CR) fluorescent stain azo dye elimination in water, all adsorption parameters were examined at room temperature. Moreover, the adsorption experiments revealed the removal capacity for uptake CR dye using BZC nanocomposite (90.14 mg g^−1^). Our results show that the BZC nanocomposite exhibited high removal capability for the adsorption of CR dye. The nanosphere adsorbent offered a simple, low-cost, and green approach for water purification and industrial wastewater control.

## Introduction

Dyes are natural and typically synthetic materials with complex aromatic structures, but stable to degrade organically. Congo red dye (CR) is toxic to many organisms which possibly produced cancer and genetic mutation. The CR dye usage is prohibited in various countries because of health issues. However, it is still widely utilized in the textile, paper, rubber, and plastic industries^1^. Different systems for removing synthetic dyes have been developed to lessen their negative environmental effects. Although there are plentiful applicable decolorization techniques, not all are effective or even appropriate for use as a result of their drawbacks. The most efficient dye removal method should be able to rapidly and essentially eliminate significant amounts of dye^2,3^. Adsorption is an extremely effective treatment for removing dissolved organic contaminants from industrial wastewater. Significant parameters that might affect adsorption efficiency such as temperature, surface area, contact time, and pH^4–6^. Numerous researchers have worked to enhance adsorption activities and make cost-effective new alternative adsorbents with high adsorption power. Recently, much emphasis placed on reaping the benefits of using nanoparticles or nanocomposites. Nanomaterials have lately received great attention in their use as adsorbents in the remediation of environmental pollutants^7^. Metal oxide nanoparticles have a higher adsorption capacity, a low diffusion resistance, a specific surface area, a faster adsorption equilibrium, and semiconducting properties. Green extract can be used to create more stable nanoparticles, can be produced in a variety of sizes and forms, and is less expensive^8–10^. The ZnO/CuO nanocomposites have garnered attention because of their outstanding controllable catalytic, optical, magnetic, and electrical characteristics, and their ecologically sound nature^11–13^. ZnO/CuO nanocomposites offer a variety of bio-uses due to their chemical, physical, and low toxicity features^14,15^. Compared to synthesis approaches that use physical and chemical processes, a green preparation of NPs is a significant and promising approach because of its numerous advantages, as the utilization of natural materials, safety, low cost, and eco-friendly^16–18^. The findings show that increasing the surface area of the nanoparticles and decreasing the energy gap through bio-fabrication of ZnO and CuO nanocomposites, either separately or together, improves the catalytic adsorption capabilities^19,20^. The biosynthesized nanocomposite demonstrated the capability to reduce dye under sunlight for removing dyes from an environment^21^. Propolis, a resinous material assembled by honeybees, is known due to its various characteristics and prospective uses^22^. It has broad-spectrum antibacterial, anti-inflammatory, and antioxidant qualities, which are useful in wound healing, dental health, skincare, and gastrointestinal health. Propolis also has noninflammatory and anticancer characteristics. Its rich bioactive content is still being explored for its different health-promoting properties, emphasizing its importance in traditional medicine and natural therapies. In this paper, ZnO, CuO, and biosynthesized ZnO/CuO (BZC) nanocomposite was biologically fabricated using propolis as bee biproduct extract. The designed nanomaterials were successfully characterized and the adsorption technique for fluorescent stain dye removal using BZC was successfully examined. The adsorption parameters were investigated, including pH, contact duration, initial concentration, and dose. The fabricated BZC nanosphere demonstrate a convenient and simple protocol for water purification and industrial wastewater management model.

## Experiments

### Materials

Zinc nitrate hexahydrate Zn(NO_3_)_2_·6H_2_O, sodium hydroxide, Sodium Carbonate Na_2_CO_3_, Ammonia(33%), copper nitrate hexahydrate Cu(NO_3_)_2_·6H_2_O and ethanol were gained from Merck (Darmstadt, Germany). The Bee byproduct (Propolis) was purchased from a domestic market (Giza, Egypt).

### Preparation of BZC using propolis extract

**Figure 1:**
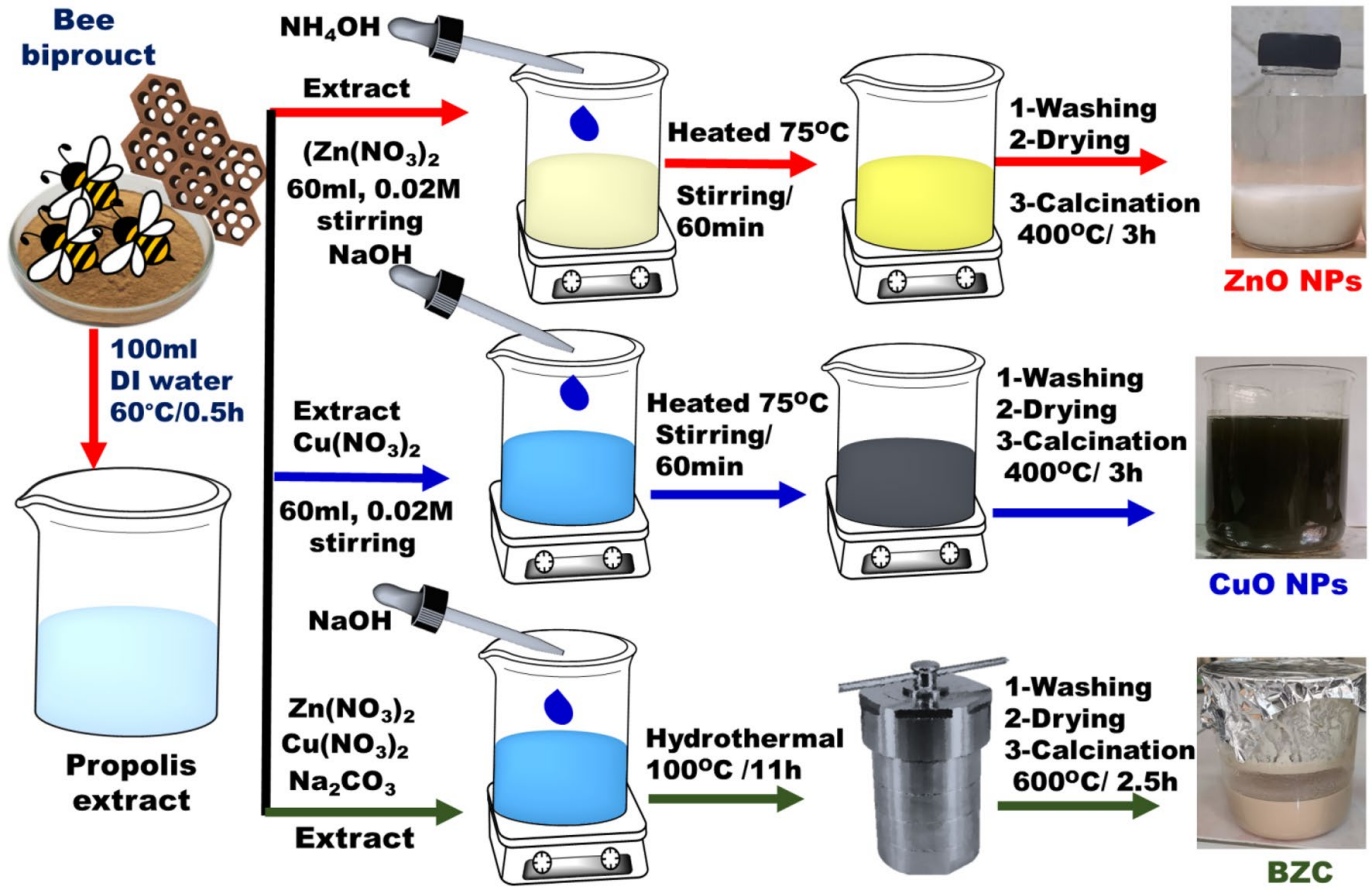
General schematic diagram for preparation of bee product extract, ZnO NPs, CuO NPs, and BZC nanocomposite.

A particular gram of propolis granules (500 mg) was dissolved into 100 ml of deionized water to produce an extract from propolis as shown in Fig. 1. To get a suitable extract, the mixture was agitated and heated to 60 °C for 30 min. After filtering, the extract was placed in a cool location to be used later. The BZC was fabricated chemically as reported^23^. Whereas for BZC nanocomposite, the same method was performed with some modification and adding propolis extract. Under continuous stirring, 50 mL of Zn(NO_3_)_2_·6H_2_O (0.1 M) were mixed with 50 mL of Cu(NO_3_)_2_·6H_2_O(0.1 M) solutions. Then, 50 mL of extract and 50 mL of 0.1 M Na_2_CO_3_ were added dropwise to the metal nitrate solutions. The obtained product was put in a stainless-steel autoclave for 11 h at 100 °C. The off-white precipitate of BZC was crushed and calcinated for 2.5 h at 600 °C (Fig. 1).

## Result and discussion

### Characterizations of nanocomposites

**Figure 2:**
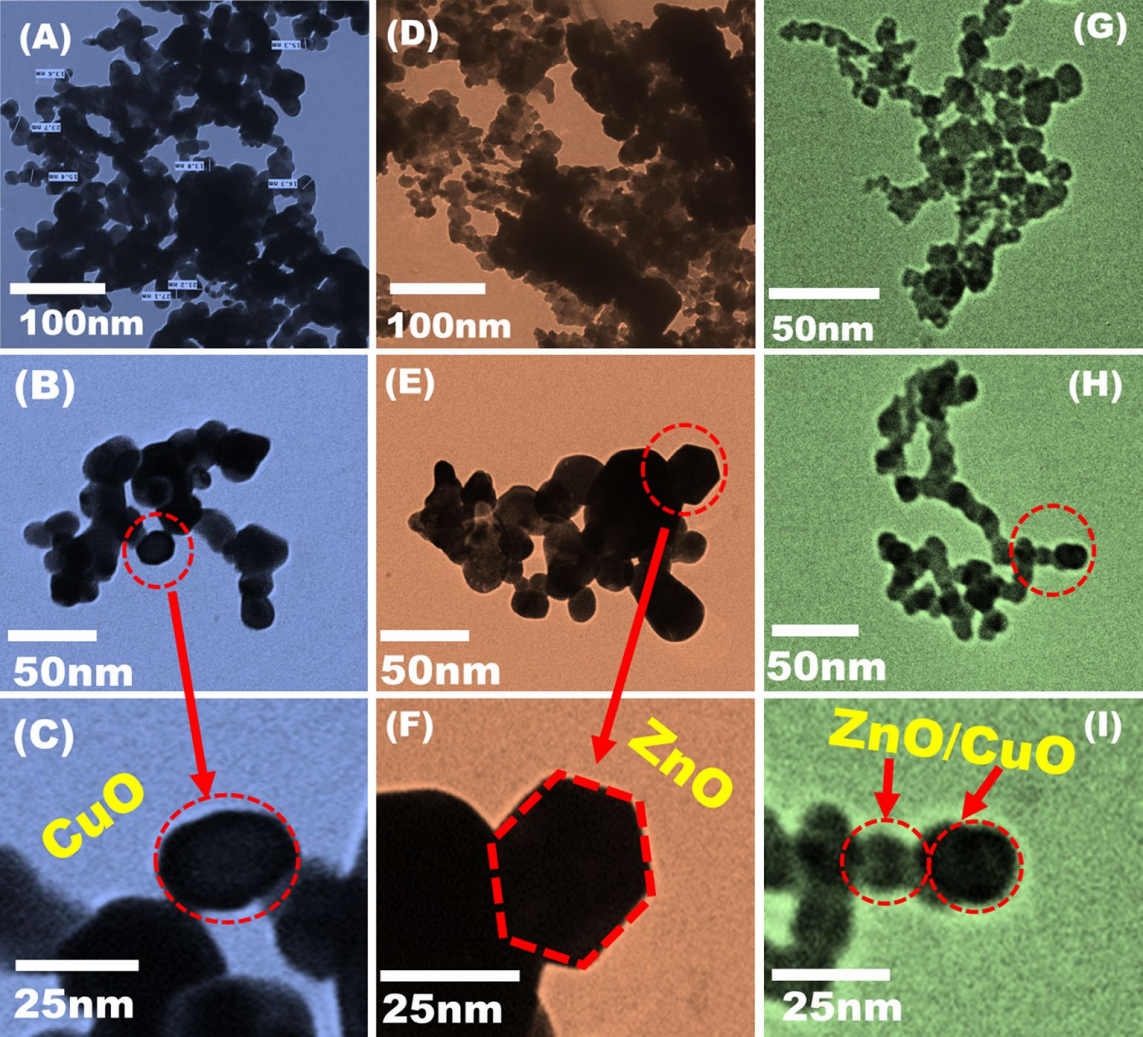
High-resolution transmission electron microscopy (HRTEM) of (**A**, **B**, **C**) CuO NPs, (**D**, **E**, **F**) ZnO NPs, and (**G**, **H**, **I**) BZC nanocomposite.

The structure features and sizes of the biologically produced BZC nanocomposites were evaluated via using TEM investigation (Fig. 2). The obtained results demonstrate that the CuO, ZnO, and BZC nanocomposites were successfully biosynthesized into nano spherical forms with different diameters ranging from 15.1 to 30 nm. The irregular shape morphology of CuO NPs with weak agglomeration was investigated Fig. 2A–C. The green synthesis of ZnO nanoparticles was nearly uniformly hexagonal shape with non-agglomerate morphology (Fig. 2D–F). An important indicator of the carefully regulated shape and molecular configurations of the dense, tight structure of BZC in necklace-like form was provided by TEM images. The condensed BZC nanocomposites appeared and related to the composition of the synthesized material Fig. 2G–I. Nanocomposite showed smaller particle sizes than pure NPs, according to TEM images. Our outcomes agreed with previous results that the produced nanocomposites varied in size from individual structures under the same manufacturing conditions^24^.

**Figure 3:**
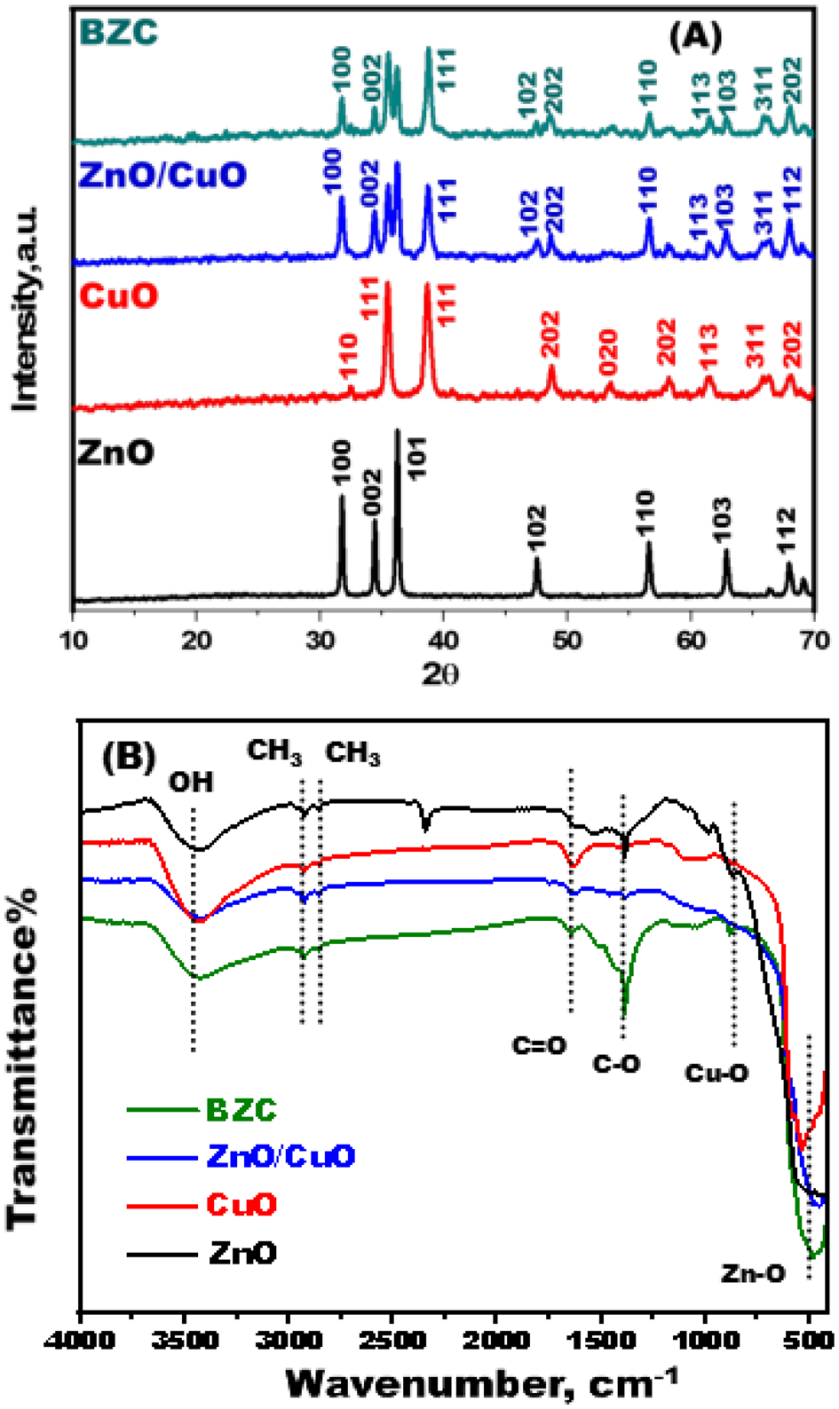
(**A**) XRD diffraction patterns of CuO-NPs, ZnO-NPs, ZnO/CuO, and the BZC adsorbents before and after removal of dye. (**B**) FTIR spectra of CuO-NPs, ZnO-NPs, ZnO/CuO, and the BZC adsorbent.

The BZC nanocomposite crystal assembly of nanoparticles was analyzed by XRD analysis. In Fig. 3A, the CuO NPs showed well-defined peaks at 32.48°, 35.7°, 38.9°, 49.2°, 53.9°, 61.8°,66.2°, 66.24° and 68.08° which agreed to (110), (− 111), (111), (− 202), (020), (-113), (022), (-311), and (220) planes respectively. The designed CuO NPs were assigned to all of the detected peaks using the JCPDS card number 01-1117^25^. The ZnO NPs main peaks were detected at 31.6°, 34.5°, 36.5°, 47.45°, 56.55°, 62.8°, and 66.14° which related to (100), (002), (101), (102), (110), (103), and (112) consistent with the JCPDS card number 5-0664 ^26,27^. The existence of ZnO and CuO peaks suggests that the BZC nanocomposites were successfully biosynthesized^28^.

FTIR analysis was used to identify the functional groups in charge of producing nanoparticles and nanocomposites (Fig. 3B). The effective synthesis of CuO and ZnO nanoparticles is responsible for the low-frequency peaks between 400 and 1000 cm^−1^^29–31^. The vibrations of stretching peaks for C–H of CH_2_ and CH_3_ groups were detected at 2862 and 2926 cm^−1^, respectively^32^. Furthermore, the peak around 1638 cm^−1^ is related to the group of the molecules from extract^33^. The peaks between 1050 and 1200 cm^−1^ are linked to C–O stretching vibrations of the extract's carboxylic acids and alcohols^34^. At 530 cm^−1^, the Zn–O bond stretching vibration emerged^35,36^. Moreover, the BZC nanocomposite spectra exhibited a peak that is ascribed to the vibrations of Zn–O and Cu–O bond.

**Figure 4:**
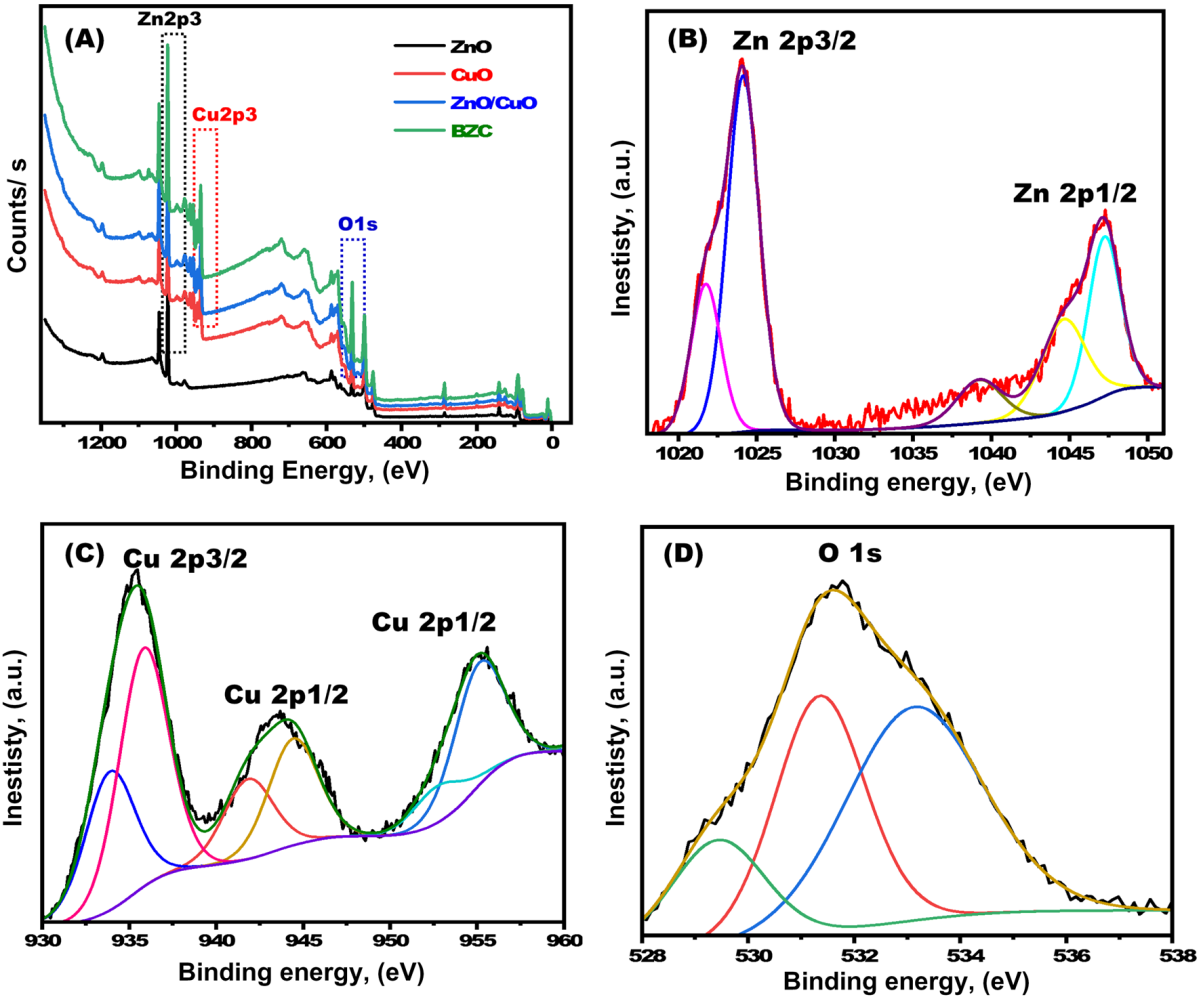
(**A**) XPS spectra survey and high-resolution spectrum of (**B**) Zn 2p, (**C**) Cu 2p, and (**D**) O 1 s of BZC architecture.

In Fig. 4, XPS was used to examine the BZC nanocomposite’s chemical composition. The Zn, O, and Cu peaks in the XPS spectra survey are the only peaks visible in Fig. 4a. The high-resolution XPS spectrum of the BZC nanocomposite's Zn2p3, Cu 2p3, and O1s were observed. The oxidation state of Zn is + 2 in the form of ZnO in the as-prepared BZC nanocomposite, according to the strong characteristic peaks centered at 1024.08 eV (Zn2p3/2) and 1047.15 eV (Zn2p1/2) from XPS spectra of Zn 2p in Fig. 4b^37^. According to the Cu 2p XPS spectra exhibited in Fig. 4c, the primary peaks are positioned at 935.18 eV and 955.285 eV, and the existence of their distinctive peaks at 941.554 eV indicates that the copper is in the + 2 oxidation state in the form of CuO^38^. The broad O1s signal (Fig. 4d), which is consisting of three smaller peaks, indicates the presence of several metal oxide states as would be predicted. A peak at 531.66 eV is attributed to the oxygen in CuO and ZnO, while peaks at 531.337 eV and 533.209 eV are assigned to oxygen adsorbed in the form of hydroxyls and carbonates. As a result, it is assumed that the O comes from oxygen adsorbed on metal oxides.

### BZC nanocomposite adsorption

**Figure 5:**
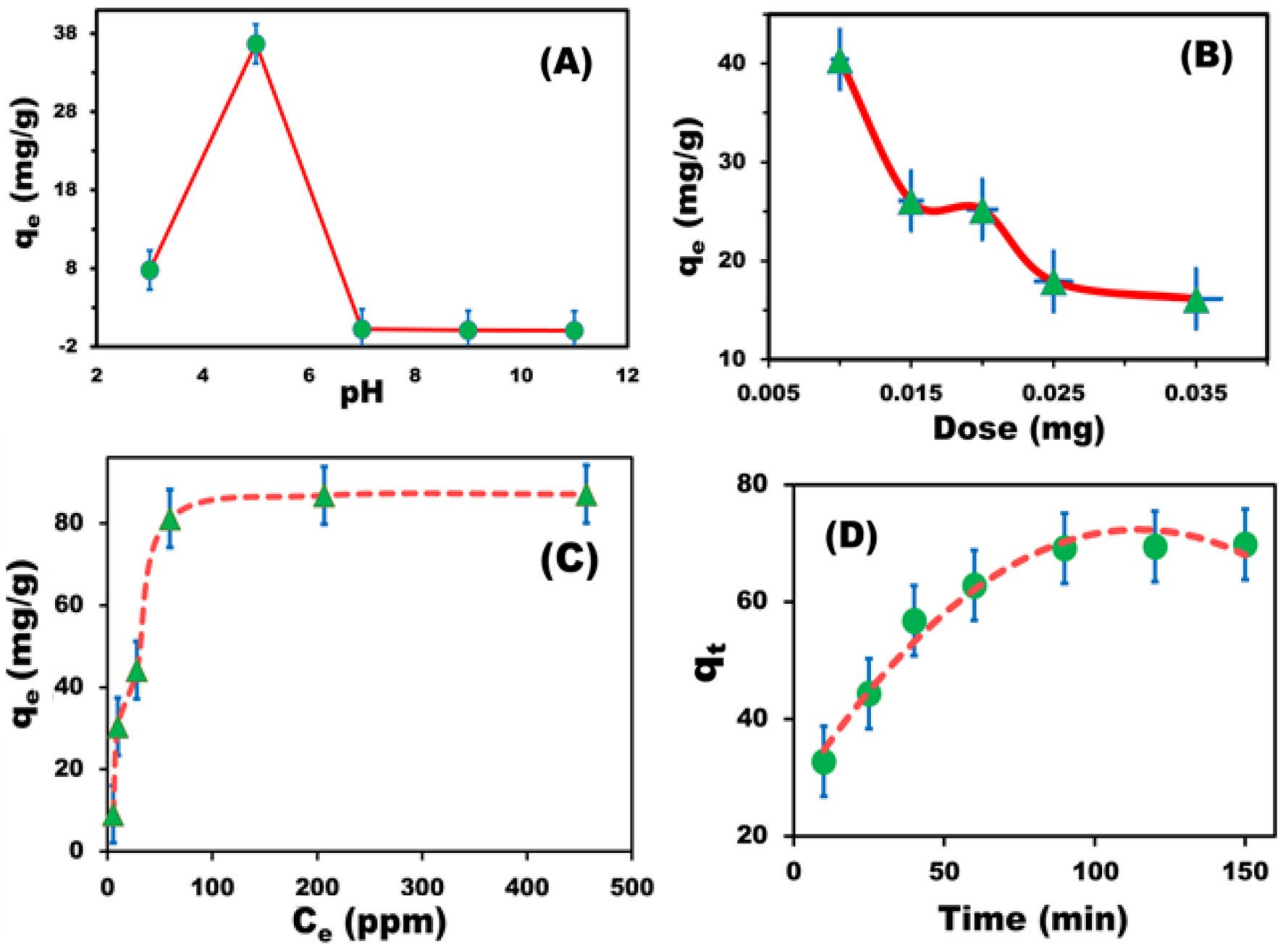
(**A**) Effect of pH on CR dye (100 mg/L) adsorption on BZC nanocomposite (0.01 g) at 25 °C. (**B**) Effect of BZC nanocomposite dosage on the adsorption capacity of the material for 100-mg/L CR dye at pH 5 and 25 °C. (**C**) Effect of CR initial concentration at pH 5 and 25 °C. (**D**) Time-dependent curves of malachite adsorption at pH 5, BZC dosage of 0.01 g, and 100-mg/L (i.e., 100 ppm) CR dye.

The optimum conditions for removing fluorescent stain dye with BZC nanocomposites were investigated. The pH variations are expected to affect CR adsorption since the solute ions' pH can affect the degree of ionization and surface charge of the BZC. The CR dye solutions ranged in pH from 2 to 12 (Fig. 5A), with the maximum adsorption efficiency occurring at pH 5. As pH elevated, the adsorption efficiency diminished. BZC surfaces seem positively charged and the solution’s positive charge on the surface interface grows at low pH, resulting in increased CR dye adsorption. Our findings revealed that the removal capacity decreased with increasing BZC dose (Fig. 5B). The initial CR dye concentrations can significantly affect adsorbent performance and the initial CR concentration was changed from 20 to 500 mg/L, and adsorption was accomplished (Fig. 5C). The dye removal percentage reduces as the original dye concentration rises, which may be because the adsorption sites on the BZC nanocomposite surface are saturated^37^. The BZC nanocomposite surface will initially have unoccupied active sites at low concentrations, and as the initial dye concentration rises, these active sites will become exhausted, and the adsorption process will not be as effective^38^. The findings of a study on the impact of contact duration on dye removal from 5 to 150 min are shown in Fig. 5D. The CR dye removal increased gradually over time until it reached equilibrium. Our findings revealed that dye elimination improved with time and reached saturation after 120 min. Our findings reveal that the CR adsorption rate is notably high within the first hour of adsorption due to the huge surface area of BZC nanocomposite and the accessibility of active centers. The CR adsorption rate then stabilized as the BZC nanocomposite pores and active sites were exhausted^39^.

**Figure 6:**
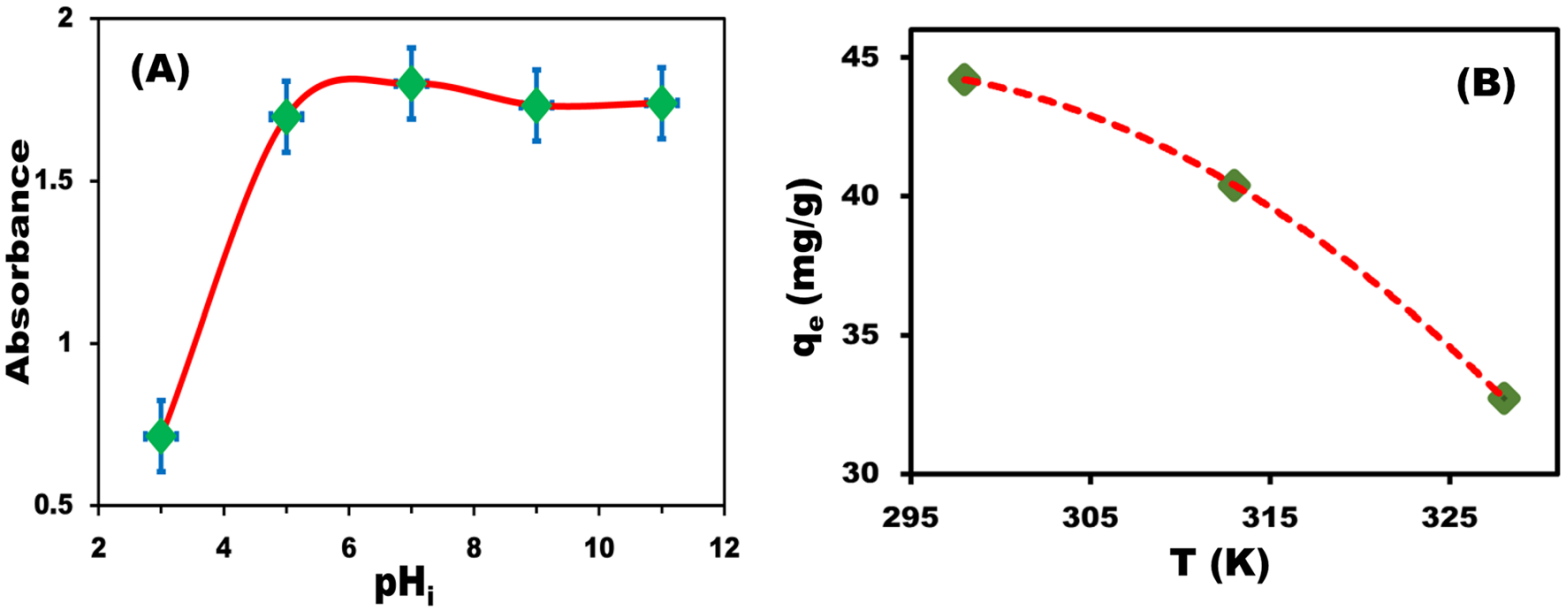
Evaluation of adsorption capacity of BZC for CR dye removal at different temperatures and optimum removal conditions.

The amount of dye adsorption (q, mg/g) onto the BZC nanocomposite decreases with increasing temperature from 25 to 55 °C (Fig. 6). Adsorption is an exothermic process, as evidenced by the reduction in adsorption capability with increasing temperature^40^. Increasing the temperature may diminish the adsorption forces between the dye and the adsorption sites on the BZC surface since the adsorption capacity is being reduced^41^.

### Adsorption isotherm

**Figure 7:**
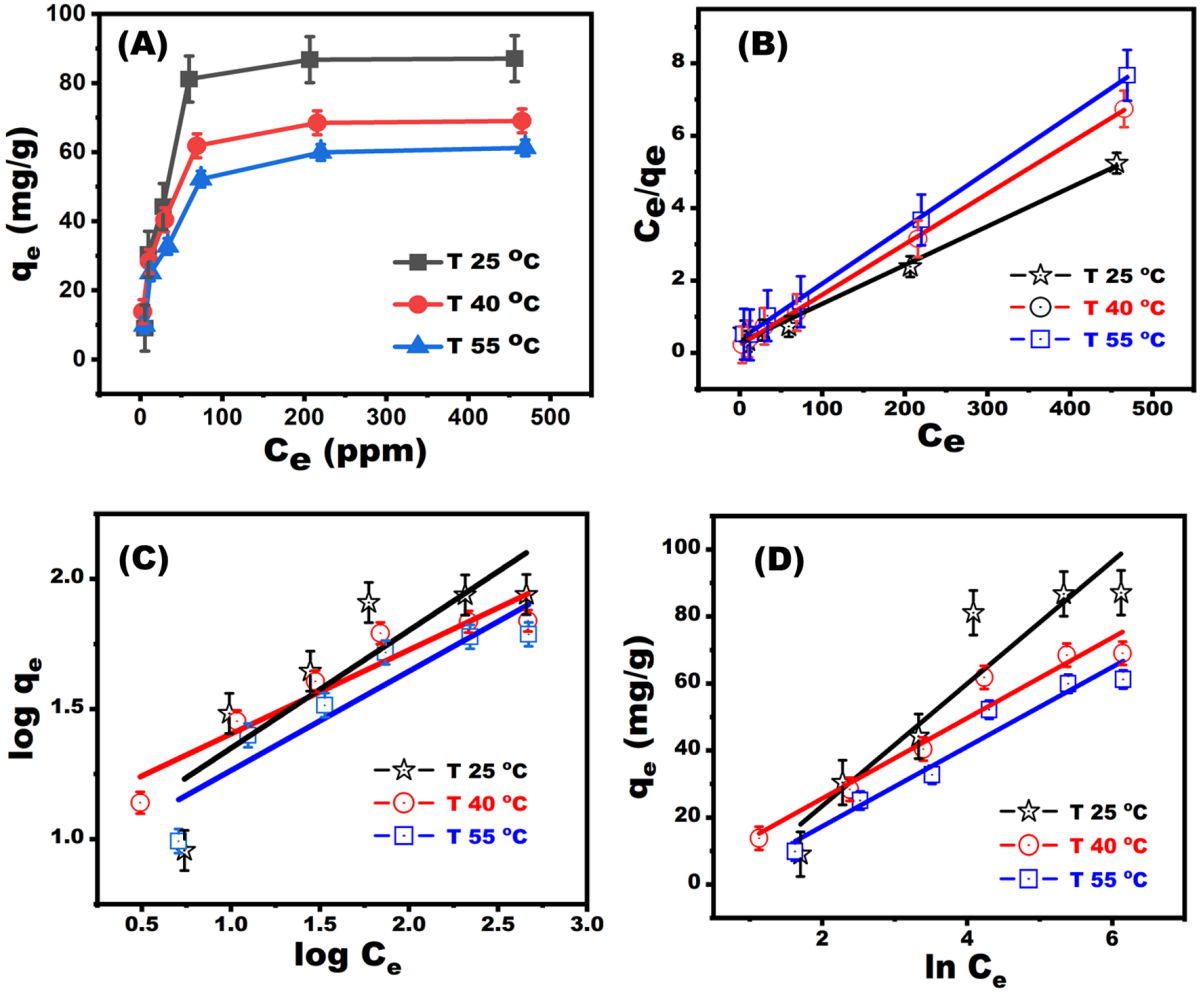
(**A**) The isotherms of removal CR dye at various temperature (**B**) the Langmuir isotherm curve, (**C**) and the Freundlich isotherm linear plots. (**D**) Trapping of CR dye on the BZC adsorbent at optimal adsorption conditions (pH 5, 0.01 g of BZC dosage, 2 h shaking time, at different temperatures).

The Langmuir, Freundlich, and Temkin models, as well as their parameters, were constructed at various temperatures to better examine the removal process (Fig. 7A). The Langmuir plot intercept and slope were evaluated at various temperatures to establish the maximal removal capacity and isotherm constant (Fig. 7B). The straight line confirmed the monolayer adsorption of dye on the BZC nanocomposites (Fig. 7B). The Langmuir isotherm is the optimal model for explaining CR dye adsorption, as further supported by the excellent correlation coefficient (R^2^ = 0.993) (Table 1). The significant adsorption and binding of the dye to the BZC cavities was further supported by the high K_L_ values observed and BZC removal capacity (q_m_) at 25 °C was 90.16 mg g^−1^, which is significantly higher than those of other adsorbents reported in Table S1. Unfavorable deviations were found after the data were fitted to the Freundlich and Temkin isotherm models (Fig. 7C,D).

**Table 1:** Isotherms and their linear forms for the adsorption of CR onto BZC nanocomposite.

Isotherm	Value of parameters			
		25 °C	40 °C	55 °C
Langmuir	q_m exp_ (mmolg^−1^)	0.13	0.1	0.09
	q_m_ (mmolg^−1^)	0.134	0.103	0.093
	K_L_ (Lmmol^−1^)	25.927	45.809	28.984
	R^2^	0.993	0.999	0.998
Freundlich	n	2.21	3.096	2.62
	K_F_ (mmoLg^−1^)(Lmmol^−1^)^1/n^	7.88	12.057	7.62
	R^2^	0.767	0.901	0.860
Temkin	b_T_(Lmol^−1^)	18.26	12.002	11.88
	A_T_ (kJmol^−1^)	0.488	1.149	0.582
	R^2^	0.887	0.946	0.949

### Adsorption kinetics

**Figure 8:**
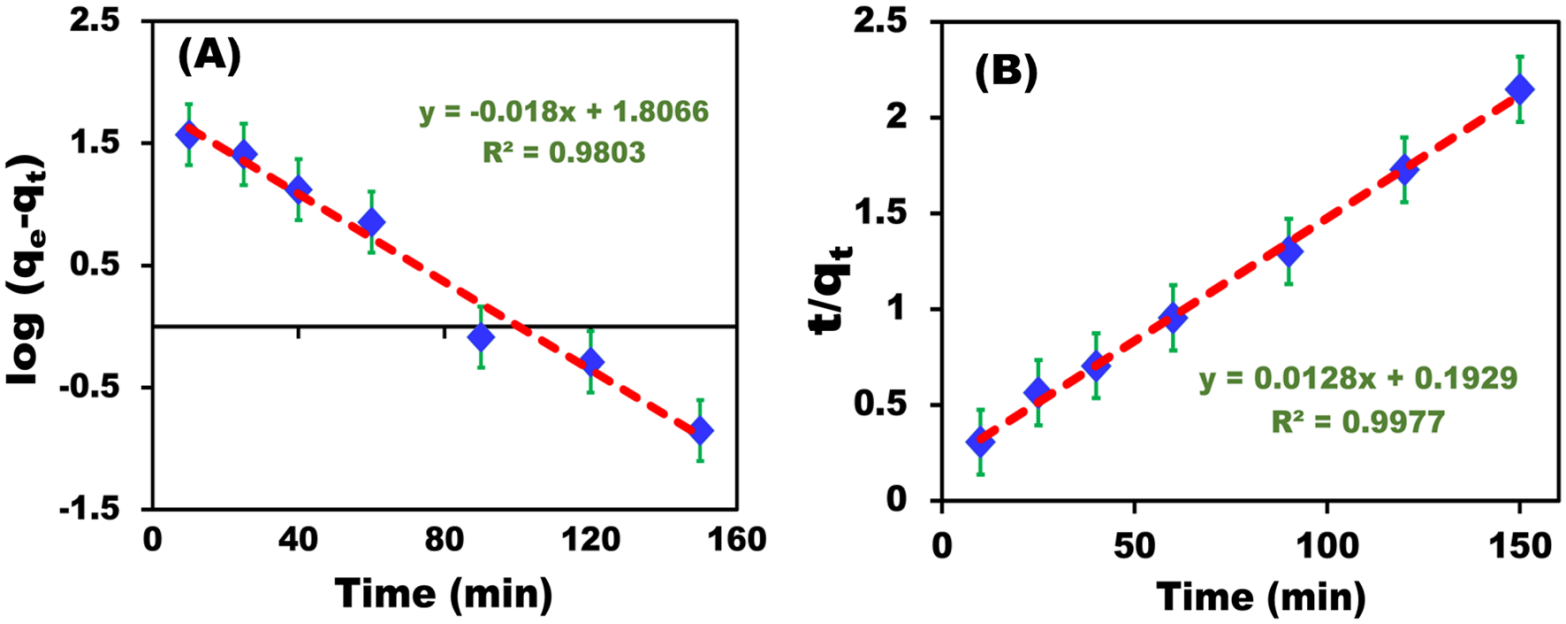
The pseudo-first order (**A**) and pseudo-second order (**B**) dye removal methods are used to evaluate the CR’s adsorption kinetics onto BZC.

In Fig. 8, the CR dye adsorption rate onto the BZC nanocomposite was initially high but decreased progressively until balance was established. After 150 min, which was considered the equilibrium time, there was a significant enhancement in the adsorption rate and maximum adsorption. The removal kinetics are critical parameters for estimating adsorption dynamics. The pseudo-first order (PFO) and pseudo-second order models (PSO) were employed to match the observations and analysis of the kinetics of CR dye removal onto BZC nanocomposites. The rate constant, K1, was determined using log (qe − qt) versus t plots, and the removal capacity at equilibrium, qe, cal, was calculated using the intercept and slope of the plot (Fig. 8A,B). The correlation coefficients obtained from the PFO were lower than the correlation coefficients obtained from PSO. Furthermore, the R^2^ values of the PSO (0.9977) for CR dye were significantly higher than those of the PFO (0.9803), implying that the dye removal kinetics follow the PSO. The correlation coefficients and q_e_ (calc.) values from the PSO are coherent with the observed data (Table 2).

**Table 2:** Kinetic parameters and their correlation coefficients for the adsorption of CR onto BZC.

Model	Value of parameters	
Pseudo-First-order kinetic	*K*_*1*_ (min^−1^)	0.018
	q_e_ (mmolg^−1^)	6.089
	R^2^	0.9803
Pseudo-second-order kinetic	*K*_*2*_ (g mg^−1^ min^−1^)	0.5931
	q_e_ (mmolg^−1^)	0.112
	R^2^	0.998
Intraparticle diffusion	K_i_ (mgg^−1^ min^1/2^)	0.2126
	C (mgg^−1^)	− 4.463
	R^2^	0.877
Experimental data	q_e_ (exp) (mmolg^−1^)	0.13

### Thermodynamic studies

**Figure 9:**
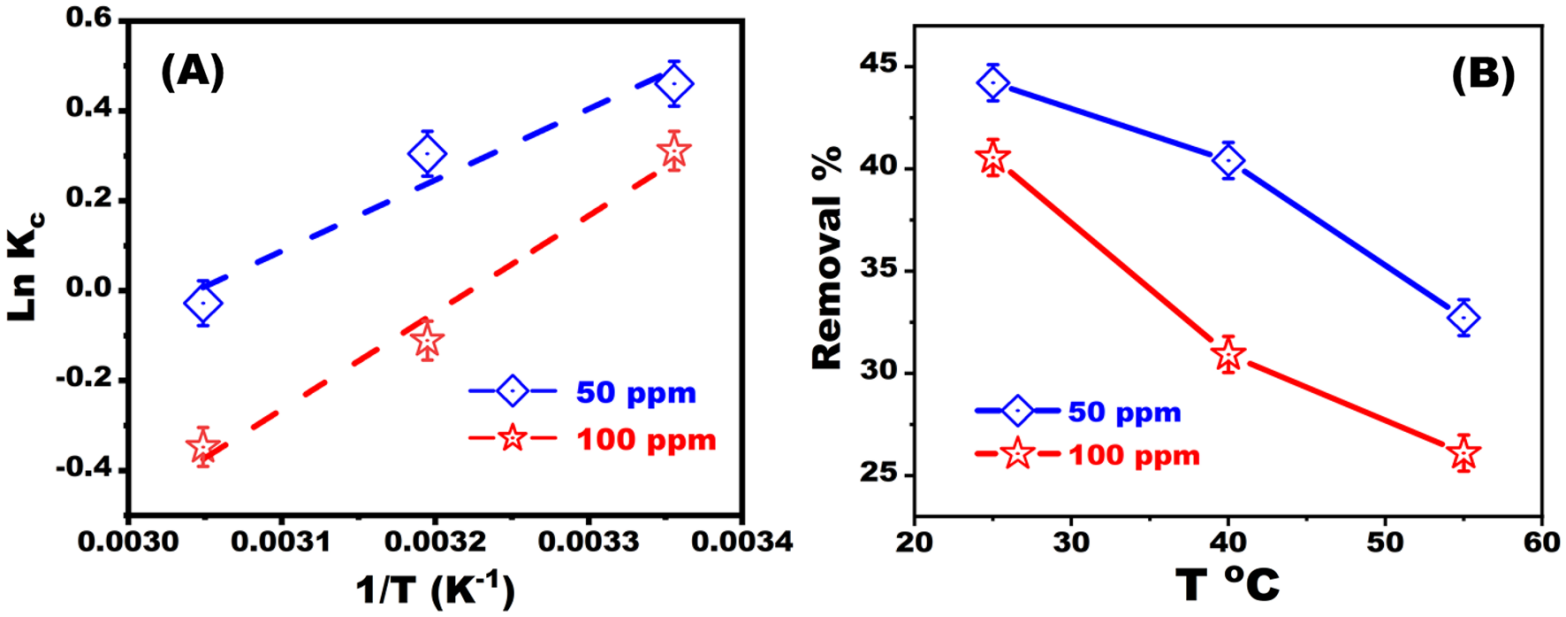
(**A**) Thermodynamic parameters at optimum conditions. (**B**) Adsorption of dye with a time dependence at different temperature intervals (25, 40, and 60 °C).

The temperature effect on the removal process is critical for practical adsorbent uses at 25 °C, 40 °C, and 60 °C (Fig. 9). The BZC nanocomposite adsorption capacity for dye was reduced when the temperature was raised from 25 to 60 °C, indicating that CR adsorption onto the BZC nanocomposite is exothermic. The calculating results of the essential thermodynamic parameters were displayed (Table 3). Physisorption changes free energy between 20 and 0 kJ mol^−1^, whereas chemisorption changes free energy between 80 and 400 kJ mol^−1^. The negative value of **∆**H and **∆**G confirms that the adsorption process is exothermic and spontaneous. It is confirmed that the reaction is more likely to occur as temperature rises by the increase in the negative value of **∆**G°. The **∆**S negative value exposed the decreased randomness (orderliness) at the BZC nanocomposite^42^.

**Table 3:** Standard enthalpy, entropy, and free energy changes for CR adsorption.

Dye	Conc ppm	∆H^o^ (kJ mol^−1^)	∆S^o^ (J mol^−1^ K^−1^)	∆G^o^ (kJ mol^−1^)		
				298 K	313 K	328 K
CR	50	− 13.14	− 39.996	− 1.2196	− 0.6197	− 0.0197
	100	− 17.91	− 57.726	− 0.7105	0.1554	1.0213

### Intraparticle mechanism

**Figure 10:**
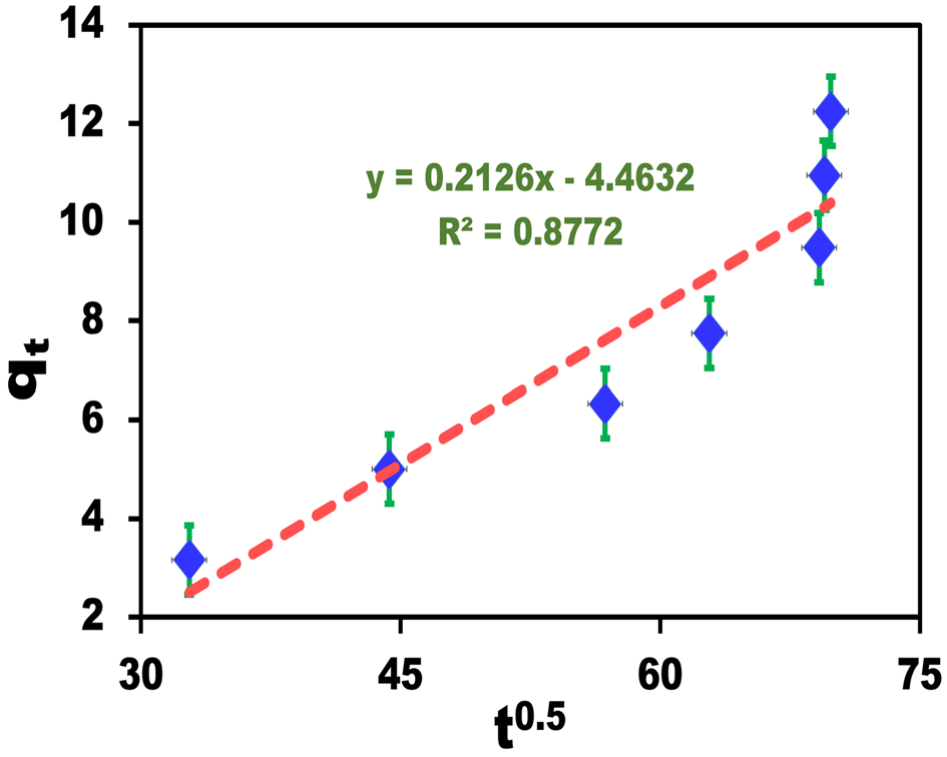
Analysis of the CR dye's uptake kinetics using a representation of intraparticle diffusion.

The dye removal using BZC nanocomposites was studied from the corresponding initial linear region slope, and the intraparticle mechanism of diffusion was demonstrated (Fig. 10). Our findings suggested that the early removal stages were reliable for controlling the mass transfer resistance to CR dye particles. The electron exchange between the BZC surfaces and the dye appears to be the rate-limiting stage in the chemisorption process. The negatively charged dye and the positively charged on the BZC nanocomposite surface could spontaneously interact to demonstrate the mechanism of adsorption. The BZC nanocomposite showed a significant capacity for CR dye adsorption in an acidic solution. The enhanced performance in an extremely acidic environment was driven through the highly charged BZC. These findings suggest that BZC is a highly applicable adsorbent for the removal of anionic dyes. The removal mechanism of CR through adsorption using BZC nanocomposite involves the physical binding of the dye molecules onto the surface of the composite material. The BZC nanosphere provides abundant active sites for CR molecules. The anionic in nature CR molecules can interact with the positively charged surface sites on the BZC composite via electrostatic forces. Additionally, there might be a chemical affinity between the functional groups on the surface of the composite material and the CR molecules which enhances the adsorption capacity. Therefore, the mechanism of CR removal using BZC nanocomposite via adsorption relies on the physical binding of the dye molecules onto the composite surface through electrostatic interactions, chemical affinity, and surface morphology, leading to the effective removal of the dye from aqueous solutions.

### Selectivity studies

**Figure 11:**
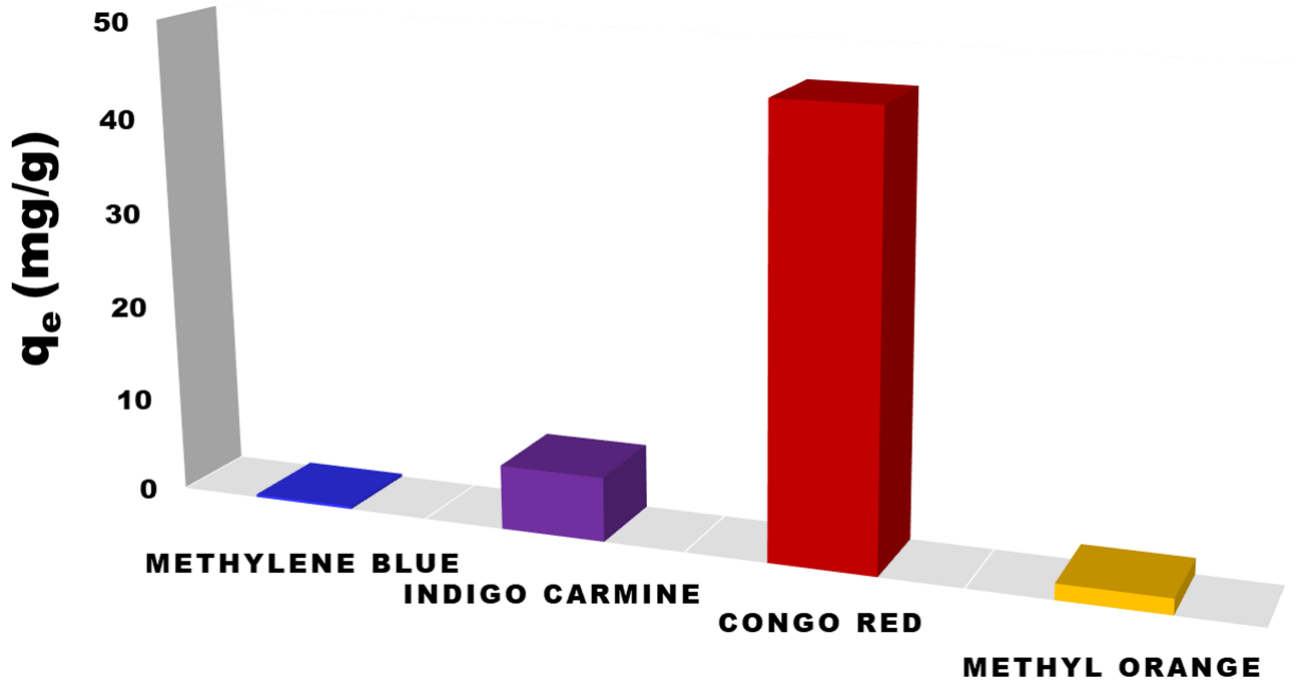
The impact of introducing different dyes at high concentrations on the trapping (uptake) mechanisms.

BZC was utilized to examine the selective trapping and capture of dye in the existence of common cationic and anionic dyes. Prior to the addition of CR dye (100 ppm), the effect of several dyes introduced at high concentration ranges on the trapping (uptake) systems was separately investigated (Fig. 11). At optimal experimental circumstances (i.e., starting concentration (10 mg/L), 0.05 g of BZC, pH of solution at 5, shaking 120 min, and temperature of 298 K), the influence of co-existing anionic and cationic dyes was examined. Our results show that there was no effect from cationic or anionic dyes in our data.

### Reusability of BZC

The reusability of the designed absorbent was investigated using a strong stripping agent such as HCl solution. After elution with 0.1 M HCl solution, the separation (collection) of dye trapped by BZC was studied. After successive adsorption–desorption cycles, we noticed a shift in BZC removal capacity. The examined BZC adsorbent (i.e., CR dye-loaded adsorbent) was regenerated by adding BZC (0.02 g) and carefully washing it with HCl (0.1 M) numerous times until the pH of the washing solution reached 7. The BZC was then rinsed many times with distilled water and the remaining colors were extracted using ethanol. The clean BZC was collected and baked at 60 °C for 4 h and the regenerated BZC nanocomposite was utilized in the removal experiment. The adsorbent's regeneration efficiency after each adsorption/desorption cycle was determined to be 95.6 percent as shown in Fig. S1. The BZC nanocomposite adsorption site blockage may be the cause of the little decrease in regeneration efficiency. Our finding is referred to as BZC cost-efficient when used in several cycles while maintaining its efficiency for dye removal, separation, and recovery after regeneration.

## Conclusion

The indirect discharge of vast volumes of toxic dyes into water has had a significant impact on the ecosystem. In this manuscript, eco-friendly BZC nanocomposites were fabricated via using the green protocol. Our results investigate the successful fabrication of ZnO/CuO nanocomposite with chain-like morphology. Moreover, the CR dye removal was studied using BZC nanocomposite under optimal adsorption process. The worm-like structure of BZC nanocomposite were accomplished for trapping CR dye. The BZC adsorbent was generated with an active positive charged surface throughout, which was greatly influenced by the adsorption conditions, such as the solution pH. Our findings show that the high removal capacity of CR dye using BZC was observed and preserved after the regeneration process. The BZC adsorbent provided a green, low-cost, and simple procedure for the removal of fluorescent azo dye for water purification and industrial wastewater control. In conclusion, there are a number of prospective applications for the adsorption of organic dyes utilizing nanomaterials. In addition to environmental remediation, other potential research areas include industrial wastewater treatment, the creation of effective dye elimination systems for the textile industry, and the production of sophisticated filtering membranes for water purification.

### Supplementary Information

Supplementary Information.

## Data Availability

All data generated or analyzed during this study are included in this published article and its supplementary information files.
