# Supplementary material for: Effective adsorption of fluorescent congo red azo dye from aqueous solution by green synthesized nanosphere ZnO/CuO composite using propolis as bee byproduct extract
Source: Sci Rep. 2024 Apr 20;14:9061. doi: 10.1038/s41598-024-58306-1 (PMC11032356; doi:10.1038/s41598-024-58306-1)
Supplement: Supplementary file 1 — Supplementary Information. [file 41598_2024_58306_MOESM1_ESM.docx]

**Supporting information**

**Effective Adsorption of Fluorescent Congo Red Azo Dye from Aqueous Solution by Green Synthesized Nanosphere ZnO/CuO Composite Using Propolis as Bee Byproduct Extract**

A. Radwan^1^, S. O. Mohamed ^2,3^, M. M.H. Khalil^1,^* I. M. El-Sewify^1,*^

^1^Department of Chemistry, Faculty of Science, Ain Shams University, 11566, Abbassia,

Cairo, Egypt.

^2^ Physics Department, Faculty of Science, Ibb University,Ibb, Yemen.

^3^ Engineering Collage, Al Janad University for Science and Technology, Taiz, Yemen.

Email: [khalil62@yahoo.com](mailto:khalil62@yahoo.com) (M.M. H. Khalil) [eslamelsewify@sci.asu.edu.eg](mailto:eslamelsewify@sci.asu.edu.eg) (I. M. El-Sewify).

**Characterization instruments**

Shimadzu Ultraviolet Spectrometer 2700 was used to study the optical properties of samples. To observe the effect of biomolecules on the reduction and capping BZC nanoparticles, Nicolet 6700 FTIR spectrometer was used. X-ray diffraction analysis of the samples was performed using a Bruker D8 Discover diffractometer equipped with a Cu microfocus X-ray source (1.5406nm) and a 2-dimensional Vantec 500 detector. The operation current and voltage were 40Kv and 40mA, respectively. The X-ray photoelectron spectroscopy (XPS) data of the samples were collected on K-Alpha (Thermo Fisher Scientific, USA) with monochromatic X-ray Al K-Alpha radiation -10 to1350 eV spot size 400micro m at pressure 10^-9^ mbar with full spectrum pass energy 200 eV and at narrow spectrum 50eV. TEM examination was performed using a JEOL GEM-1010 transmission electron microscope with a 70 kV accelerating voltage to determine the shape and size of the nanoparticles. A drop of a particle-containing liquid was dropped onto a copper grid and kept at room temperature by permitting water evaporates. Excited state lifetimes were measured with Easylife from OBB using 375nm LED as an excitation light source.The pH values of solutions were measured using a pH-meter (HANNA INSTRUMENTS PH211 Microprocessor, Romania).

**Preparation of ZnO nanoparticles**

To synthesis ZnO nanoparticles, 0.2M Zinc nitrate hexahydrate [Zn(NO_3_)_2_.6H_2_O]. Then 60 ml of the prepared solution was mixed and stirred magnetically with 20 ml of the propolis aqueous extract solution. The pH of the mixture was adjusted to 7 using drops of Ammonia (33%). The mixture solution was heated to 75 ^0^C for one hour and a white-yellowish precipitate was formed. The precipitate was then gathered by centrifuging and rinsing with deionized water and ethanol to remove excess reagents. Eventually, the ZnO nanoparticles precipitate was dried at 100 ^O^C for 6 hours and calcinated at 400^o^C for 3 hours till it became a white precipitate.

**Preparation of CuO nanoparticles:**

For synthesizing CuO nanoparticles, 0.2M of Cu(NO_3_)_2_.6H_2_O was prepared. Then 40 ml of the prepared solution was mixed and stirred magnetically with 20 ml of the propolis aqueous extract preparation. The mixture pH was adjusted to 8 using drops of sodium hydroxide solution. The mixture was heated to 70 ^0^C for one hour and a precipitate was formed. The mixture was centrifuged and washed with deionized water and ethanol. The precipitate (CuO nanoparticles) was dried using an oven heated to 100 ^o^C for 6 hours and then calcinated at 400^o^C for 3 hours.

**Adsorption studies**

A series of experiments were designed to the adsorption properties of BZC nanocomposites. For this purpose, specific milligrams of BZC nanocomposites were added in 20 mL Congo Red dye aqueous solution, with specific concentration(mg/L), in different beakers under continuous shaking for 3 hours. The absorbance of Congo red dye was measured at different pH values (2, 5, 7, 9, 11). Then, 3 ml of the solution was collected every at 15 minutes time interval and centrifuged to remove the suspended particles. The absorbance of Congo red dye was evaluated using a UV–vis spectrophotometer at 498 nm before and after the addition of BZC nanocomposite. The percentage removal of CR was calculated by:

$$Removal \%=100\%\times\frac{\left( C_{o}-C_{e} \right)}{C_{o}}$$

where C_o_ and C_e_ represent initial and equilibrium Congo Red dye concentrations (mg/L) aqueous solution, respectively.

The following formula is used to calculate the equilibrium adsorption capacity of adsorbents (qe):

$$q_{e}=\left( C_{o}-C_{e} \right)\times\frac{V}{m}$$

where *V* and *m* represent the the volume of Congo Red dye (L) and the mass of adsorbent (g), respectively.

The isotherm and thermodynamic studies were conducted by looking at how temperature (30–60 °C) affected adsorbent doses (5mg–25mg) in 50 mg/l of CR. The kinetic study was carried out at room temperature (30 °C) by varying the concentration (10–250 mg/l) and time (0–180 min). Following that, the various parameters of isotherm, kinetic, and thermodynamic studies were examined.

Three models, including the Langmuir, Freundlich, and Temkin isotherms, were used to analyze the data in order to further investigate the adsorption capacities of the nanocomposites.

**2.7. The pH point of zero charge estimation**

The pH of point of zero charge (PZC) was investigated using the pH drift method[30, 31]. pHPZC was determined by keeping 120 ml of a 0.01M KCl solution in a 150ml beaker. Then, 20 ml was poured into 6 beakers. The pH of each beaker was then adjusted to a consecutive integer ranging from 2 to 12, using a micropipette to carefully add the required amount of either 0.1M HCl or 0.1M KOH. The powdered BZC nanocomposite 20mg samples were added to the solution, capped, and shaken. To calculate pH_PZC,_ the final pH of all BZC samples was measured after 48 hours and plotted against the initial pH. The pH at which the curve crosses the pH initial = pH final line is regarded as the PZC of the corresponding BZC nanocomposites.

**Figure S1.** The adsorption and regeneration of BZC for multiple cycles where the stripping agent was 0.1 M HCl.

Table S1. Comparison of adsorption of proposed work and others

| Adsorbent | Q_m_ (mg/g) | Reference |
| --- | --- | --- |
| [cellulose/chitosan hydrogel beads](https://www.tandfonline.com/doi/abs/10.1080/19443994.2015.1082945) | 40 | 1 |
| [ZnO-modified SiO_2_](https://www.sciencedirect.com/science/article/pii/S0167732217340576?casa_token=0AFeuCMKPzcAAAAA:ya0WtDJj52FkRHoPAdBS5zKgxiStrCHQoxUsDTpoaN1x2HwIicdQUotbBRQ2y0yp6yQ2r9nDjbgx) | 83 | 2 |
| [Crosslinked cellulose dialdehyde](https://www.sciencedirect.com/science/article/pii/S2213343716300082?casa_token=AnChOPG50mUAAAAA:wPMf_OMMWrfsyQhE9bRub2_1wAYVRuwe5RH1Ub7N990fOZa_GLg9_7PM2oKOqrrD71t12T9iQZGd) | 34.7 | 3 |
| partially oxidized graphite nanoparticles (POG-NPs) | 50 | 4 |
| [ZnO nanoparticles using Eucalyptus](https://www.sciencedirect.com/science/article/pii/S0045653519330449?casa_token=TkwGCcKDgi8AAAAA:G0qqw0lVJKoy_4aELSHuKf7H_cKuX0-f5vjcDKtqkwPoNa_8bYs8C-gzemegCaBcfaMF4k7PLOpd) | 48.3 | 5 |
| [FeNi_3_/SiO_2_/CuS](https://www.tandfonline.com/doi/abs/10.1080/03067319.2020.1754810) | 42.73 | 6 |
| zinc oxide nanoparticles using Tulsi leaf | 74.4 | 7 |
| BZC | 90.14 | Proposed work |

**References**

1. Li, Manfeng, Zhaomei Wang, and Bingjie Li. "Adsorption behaviour of congo red by cellulose/chitosan hydrogel beads regenerated from ionic liquid." Desalination and Water Treatment 57, no. 36 (2016): 16970-16980.
2. Zhang, Jiajin, Xinlong Yan, Mengqing Hu, Xiaoyan Hu, and Min Zhou. "Adsorption of Congo red from aqueous solution using ZnO-modified SiO2 nanospheres with rough surfaces." Journal of Molecular Liquids 249 (2018): 772-778.
3. Kumari, Sapana, Deepika Mankotia, and Ghanshyam S. Chauhan. "Crosslinked cellulose dialdehyde for Congo red removal from its aqueous solutions." Journal of environmental chemical engineering 4, no. 1 (2016): 1126-1136.
4. Mahmoud, Mohamed E., Mohamed F. Amira, Seleim M. Seleim, and Magda E. Abouelanwar. "In situ microwave-assisted oxidation of graphite into partially oxidized graphite nanoparticles for microwave-sorptive removal of anionic and cationic dyes." *Journal of Molecular Liquids* 288 (2019): 110979.
5. Chauhan, Amit Kumar, Navish Kataria, and V. K. Garg. "Green fabrication of ZnO nanoparticles using Eucalyptus spp. leaves extract and their application in wastewater remediation." Chemosphere 247 (2020): 125803.
6. Nasseh, N., Arghavan, F.S., Rodriguez-Couto, S. and Hossein Panahi, A., 2022. Synthesis of FeNi3/SiO2/CuS magnetic nano-composite as a novel adsorbent for Congo Red dye removal. International Journal of Environmental Analytical Chemistry, 102(10), pp.2342-2362.
7. Nayak, Ansuman, Jitendra Kumar Sahoo, Shraban Kumar Sahoo, and Duryodhan Sahu. "Removal of congo red dye from aqueous solution using zinc oxide nanoparticles synthesised from Ocimum sanctum (Tulsi leaf): a green approach." International Journal of Environmental Analytical Chemistry 102, no. 19 (2022): 7889-7910.
